# Supplementary material for: Enhancing chemotherapy response through augmented synthetic lethality by co-targeting nucleotide excision repair and cell-cycle checkpoints
Source: Nat Commun. 2020 Aug 17;11:4124. doi: 10.1038/s41467-020-17958-z (PMC7431578; doi:10.1038/s41467-020-17958-z)
Supplement: Supplementary file 1 — Supplementary Information [file 41467_2020_17958_MOESM1_ESM.pdf]

SUPPLEMENTARY INFORMATION

“Enhancing Chemotherapy Response Through Augmented Synthetic Lethality by Co-Targeting Nucleotide Excision Repair and Cell-Cycle Checkpoint”

Kong et al.

1) Supplementary Figures
2) Full Western Blots

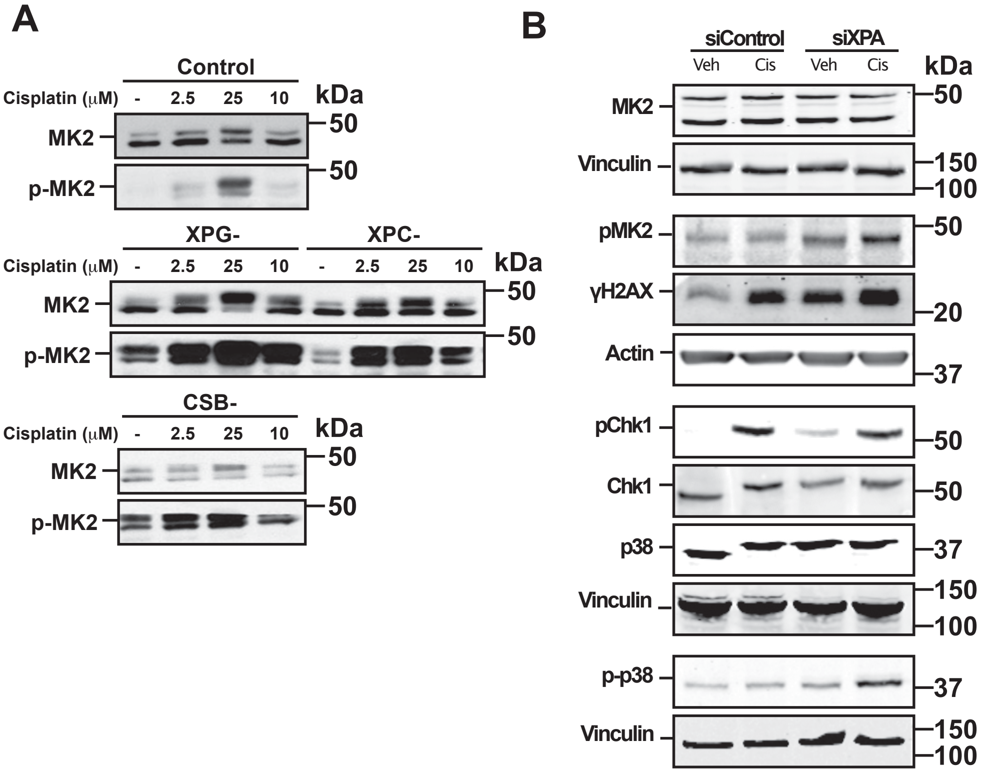

Supplementary Figure 1. NER defective cells hyperactivate MK2 signaling (Related to Figure 1). (**A**) MK2 activity as assessed by Western blot analysis 24 h after cisplatin treatment in control and NER-deficient XP and CS fibroblasts. n=2 independent experiments. The blot from control fibroblasts is reproduced from Figure 1A for comparison, since the experiments shown here and in Figure 1A were performed together. (**B**) Loading controls for western blots shown in Figure 1. n=3 independent experiments.

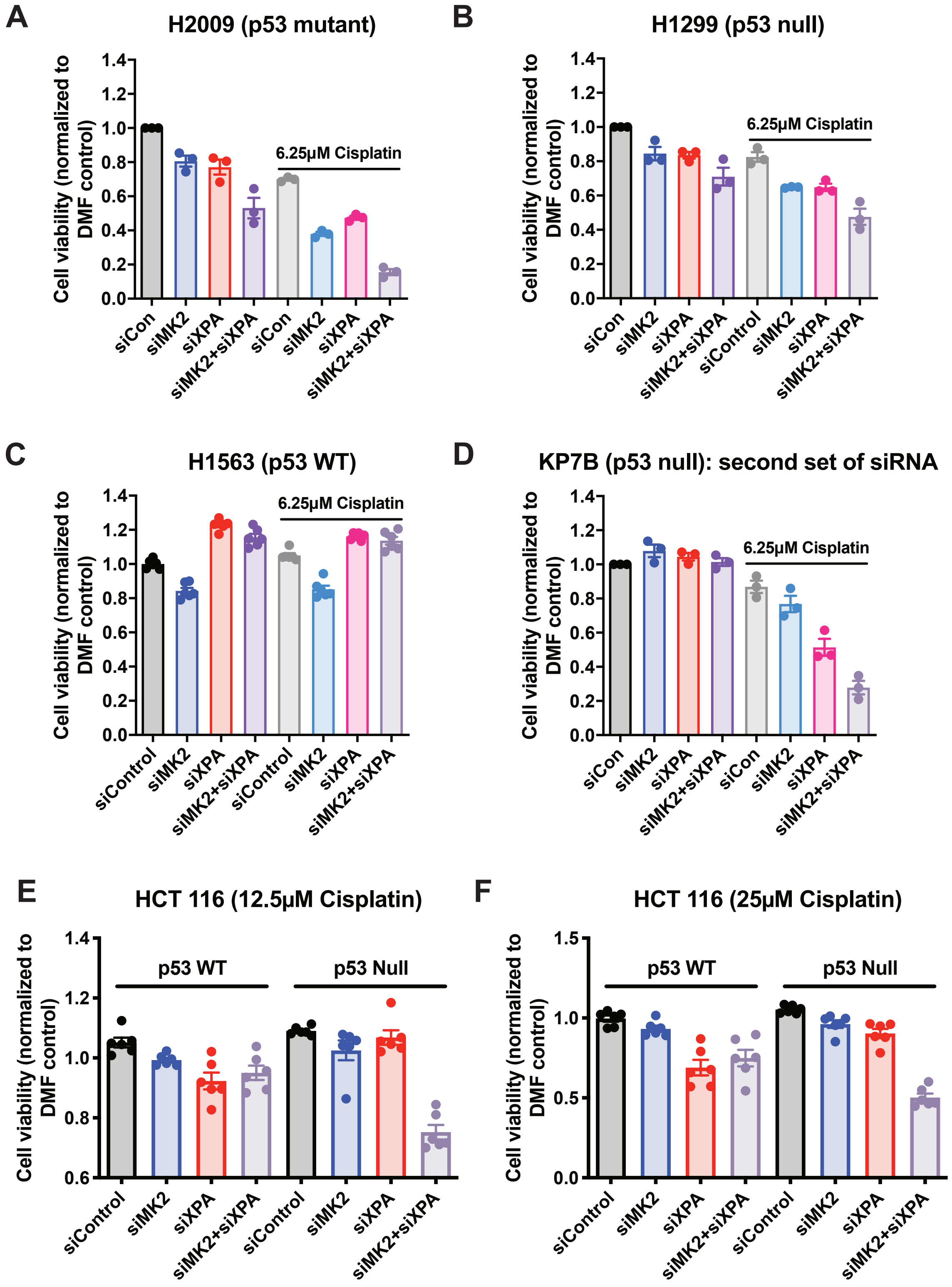

Supplementary Figure 2. Combinatorial targeting of XPA and MK2 enhances cisplatin lethality in cells. (Related to Figure 2). A-C) Cell viability assays were performed in (**A**) H2009 (n=3), (**B**) H1299 (n=3), and (**C**) H1563 (n=6) cells depleted of either MK2, XPA, or both MK2, and treated with cisplatin *in vitro.* (**D**) Cell viability assay in KP7B cells (n=3) using a second set of siRNAs against MK2 and XPA and treated with cisplatin *in vitro.* (**E-F**) Cell viability assay in HCT116 p53 wild type and p53 null isogenic cells (n=6) depleted of either MK2, XPA, or both MK2, and treated with (**E**) 12.5M or (**F**) 25M cisplatin *in vitro.* Note the enhanced lethality of cisplatin in MK2/XPA doubly-depleted cells compared with singly depleted cells in the p53 mutant cell lines, but not in the p53 wild-type cell line. In all panels, error bars represent mean+/- SEM.

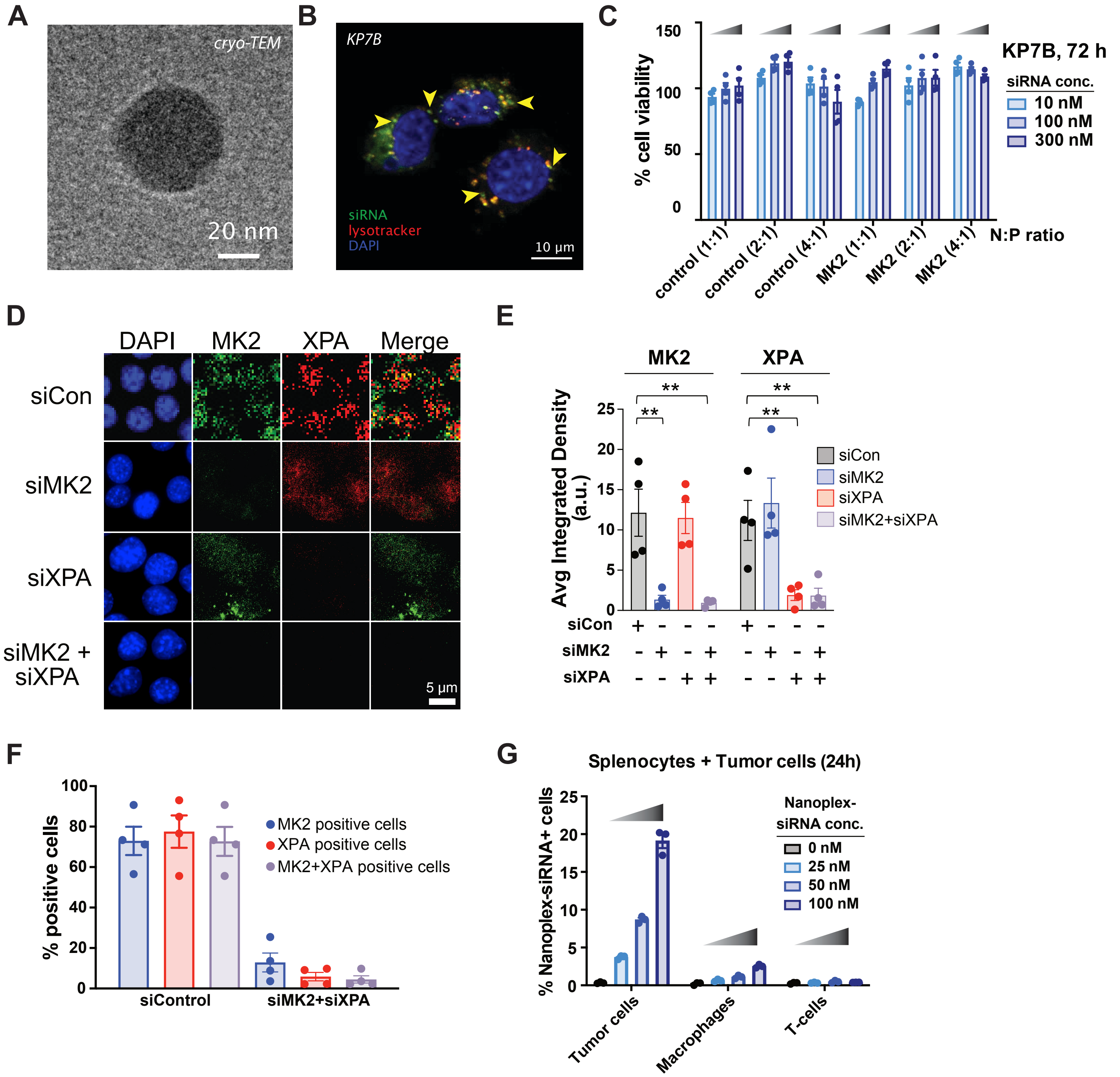

Supplementary Figure 3. Nanoplex delivery of functional siRNAs to NSCLC cells. (**A**) Cryo-electron microscope image of nanoplexes. (**B**) Nanoplexes deliver siRNA to cytoplasm of NSCLC cells. Yellow arrows indicate localization of nanoplexes. Panels A and B representative of n=3 samples. (**C**) Cell viability of KP7B cells was not affected by nanoplex-siRNA. Cell viability was assessed by ATP content (CellTiter-Glo) 72 hrs after transfection with the indicated concentrations of siRNA and varying ratios of polymer amine to siRNA (N:P ratio). (**D**) Representative immunofluorescence images of KP7B cells depleted of MK2, XPA, or both, stained with an antibody against MK2 or XPA showing co-depletion within individual cells. (**E**) Average integrated density for either MK2 or XPA staining in the KP7B cells shown in Fig. S3D. MK2 staining: siMK2 vs siCon, ** p=0.0018; siMK2+siXPA vs siCon, ** p=0.0012. XPA staining: siXPA vs siCon, ** p=0.0071; siMK2+siXPA vs siCon, ** p=0.0067. 2-way ANOVA, p=adjusted p value. (**F**) Quantification of MK2, XPA or MK2/XPA dual-stained KP7B cells following treatment with nanoplex-siControl or nanoplex-siMK2/XPA. Data in panels C-F represent n=4 separate samples. (**G**) Nanoplex siRNAs are preferentially taken up by tumor cells. $5x10^5$ primary mouse splenocytes were co-cultured with an equal number of KP7B tumor cells. The co-culture was treated with fluorescent siRNA-containing nanoparticles and the percentage of fluorescent cells for each cell type was quantified by FACS analysis using surface markers. Data represents 3 separate samples. Error bars in panels C and E-G represent mean+/- SEM.

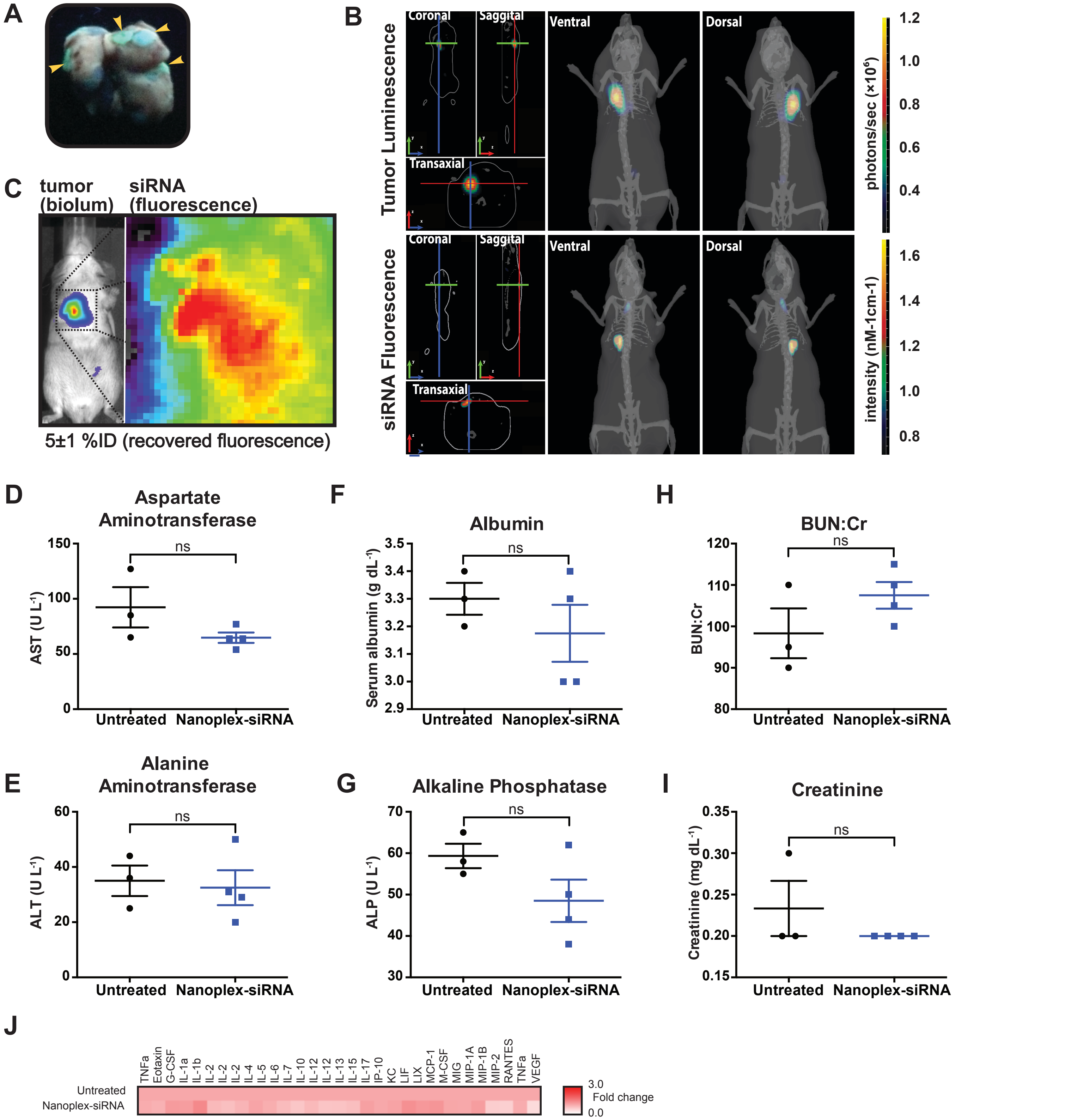

Supplementary Figure 4. siRNA Nanoplex particles partition into NSCLC tumors. (**A**) Example of GFP-labeled tumors (yellow arrows) from control nanoplex-treated animals at necropsy. (**B**) Representative images showing accumulation of nanoplex-siRNA in lung tumors. Upper panel shows bio-luminescence of lung tumor. Lower panel shows siRNA fluorescence localizing to lung tumors. (**C**) Close up image showing accumulation of nanoplex-siRNA in lung tumor. Left panel shows bioluminescence of lung tumor. Right panel shows accumulation of siRNA fluorescence localizing to lung tumors. (**D-I**) Liver and kidney toxicity markers; Levels of (**D**) Aspartate Aminotransferase, (**E**) Alanine Aminotransferase, (**F**) Albumin, (**G**) Alkaline Phosphatase, (**H**) BUN:Cr, and (**I**) Creatinine were measured in serum from n=3 for untreated and n=4 for nanoplex-siRNA treated animals. (**J**) Serum cytokine profiles. Data are shown as fold-change vs. the untreated control from n=3 animals in each group. No statistically significant differences were noted for any cytokine. In panels D-J, nanoplex siRNA-treated animals received 1mg kg^{-1} siRNA encapsulated with 200 mg kg^{-1} nanoplexes. Error bars in panels D-I represent mean+/-SEM. ns indicates not significant; two-tailed unpaired t-test.

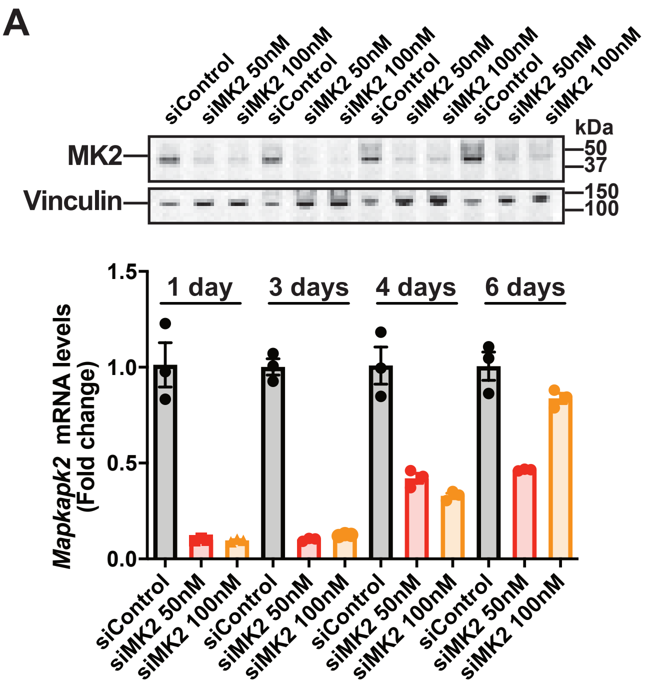

Supplementary Figure 5. Time course showing maximal MK2 knockdown at 3 days. (**A**) MK2 protein levels assayed by Western blot (top) and RNA levels assayed by RT-qPCR (bottom) were measured in KP7B cells at various time points after siRNA transfection. Data show mRNA levels as fold-change vs. the untreated control and western blots showing MK2 protein levels at times post siRNA transfection. n=3 samples, error bars represent mean+/- SEM.

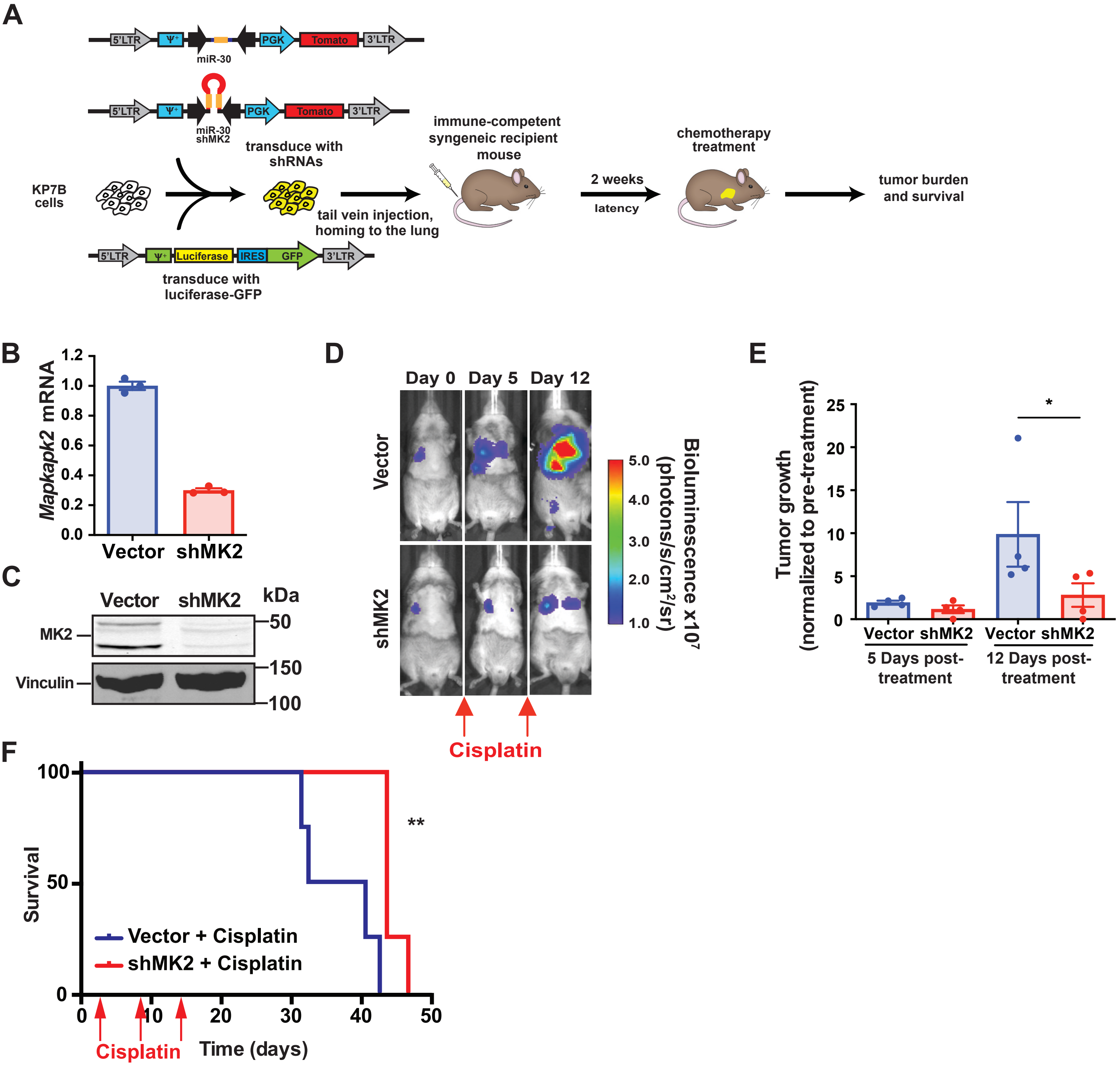

Supplementary Figure 6. MK2 knock down enhances anti-tumor response to cisplatin. A) Schematic representation of the transplantable model. KP7B cells are transduced with a vector expressing luciferase-GFP and a vector expressing hairpin against MK2. Schematic modified from Cannell *et al* [18]. (**B**) RT-qPCR levels of MK2 mRNA in control and MK2 shRNA stable knockdown KP7B cells. (**C**) Western blot for MK2 from KP7B cells in panel 2B. (**D**) Representative bioluminescence images before and after cisplatin treatment on days 0, 5 and 12. Red arrows indicate timing of cisplatin dosing. (**E**) Quantification of lung bioluminescence 5 and 12 days post-cisplatin treatment. Error bars represent mean+/- SEM, four animals per condition. * p=0.0354; one-way ANOVA. (**F**) Kaplan-Meier survival analysis of control or shMK2 tumor-bearing mice, post cisplatin treatment, as indicated. Red arrows indicate timing of cisplatin dosing. (Vector + Cis n=5, shMK2 + Cis n=4; ** p=0.0067). p values were calculated using the log-rank test.

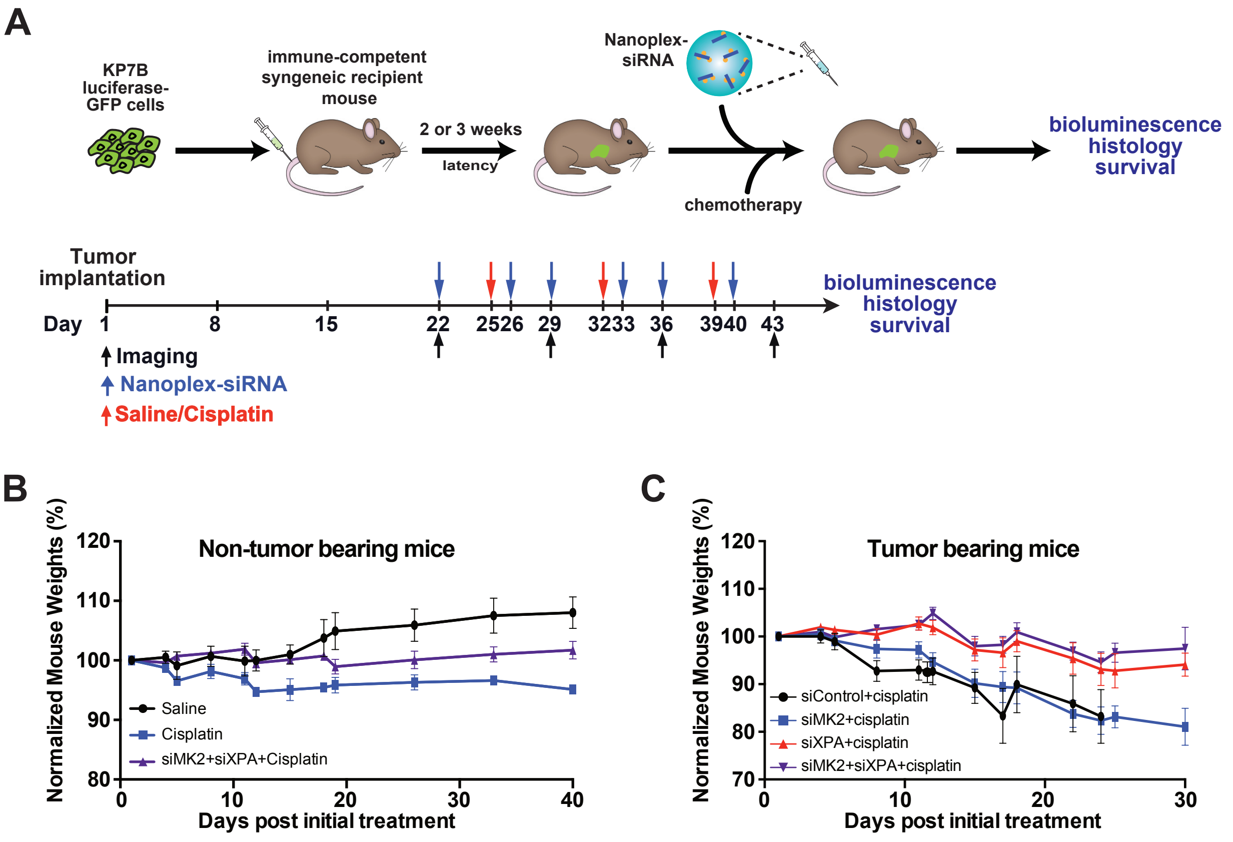

Supplementary Figure 7. siRNA Nanoplexes plus cisplatin show limited toxicity *in vivo.* (A) Timeline of combination nanoplex-MK2/XPA siRNA and cisplatin treatment of mice with tumors. Blue arrows indicate time of nanoplex-siRNA treatment. Red arrows indicate time of cisplatin treatment. **(B)** Weights of non-tumor-bearing mice treated with saline, cisplatin alone and nanoplex-siMK2/XPA in combination of cisplatin. n=5 animals for each condition. (**C**) Weights of tumor-bearing mice treated with nanoplex-siCon, nanoplex-siMK2, nanoplex-siXPA, and nanoplex-siMK2/XPA in combination of cisplatin

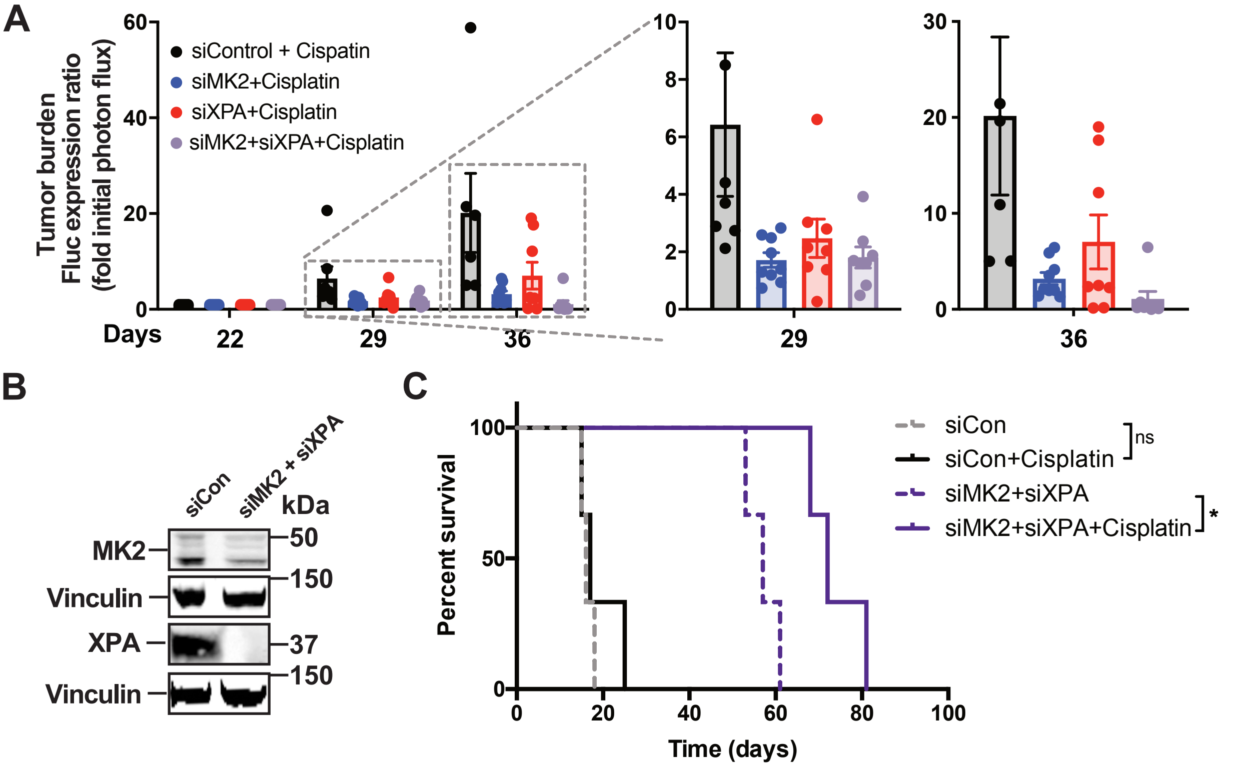

Supplementary Figure 8. Pt treatment with co-targeting of XPA and MK2 enhances survival (Related to Figure 4). (**A**) Quantification of lung bioluminescence 29 (1st treatment) and 36 (2nd treatment) days after tumors develop. Data shows bioluminescence as fold-change compared to pre-treatment on day 22 (nanoplex-siControl + cisplatin n= 7 animals, nanoplex-siMK2 + cisplatin n= 9 animals, nanoplex-siXPA + cisplatin n= 8 animals, and nanoplexes-siMK2/siXPA + cisplatin n= 8 animals). Error bars represent mean+/- SEM. (**B**) Representative western blot of lung tumors from a mouse harvested on day 43 following treatment nanoplex-siMK2/XPA versus those from a mouse following treatment with nanoplexes-siCon. See timeline in Supplementary Figure S7A for details. n= 3 animals. **C**) Kaplan-Meier survival analysis of tumor-bearing mice treated with the indicated treatment. n=3 animals each for the nanoplex-siControl with or without cisplatin treatment, n= 3 animals for nanoplex-siMK2/siXPA alone, and n= 5 animals for nanoplex-siMK2/siXPA + cisplatin. Addition of cisplatin to nanoplex-siMK2/siXPA increased the median survival by an additional 15 days. * $p<0.05$ calculated using the log-rank test.

A

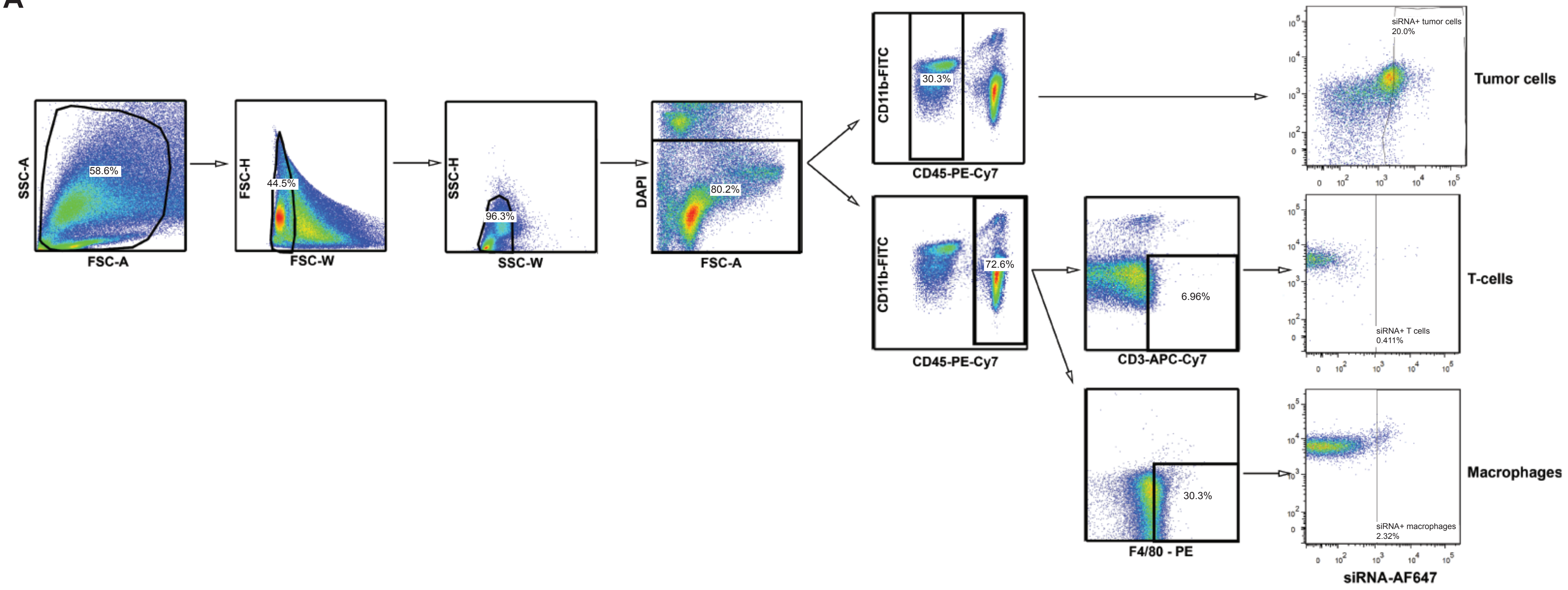

Supplementary Figure S9. Gating strategy for FACS analysis A) Gating strategy for FACS analysis of nanoplex-siRNA uptake by tumor cells, T-cells, and macrophages as shown in Supplementary Figure S3G. Primary mouse splenocytes were co-cultured with an equal number of KP7B tumor cells and treated with fluorescent siRNA-containing nanoparticles and the percentage of fluorescent cells for each cell type was quantified by FACS analysis using surface markers. Viable cells were assessed by flow cytometry for uptake of fluorescent siRNA by specific immune populations by co-staining with fluorophore conjugated antibodies for CD45, CD11b, F480 and CD3. CD45-AF647+ cells were scored as siRNA+ tumor cells, CD45+CD11b+AF647+ cells were scored as siRNA+ macrophages, and CD45+CD3+AF647+ cells were scored as siRNA+ T-cells.

Figure 1B and Supplementary Figure 1A

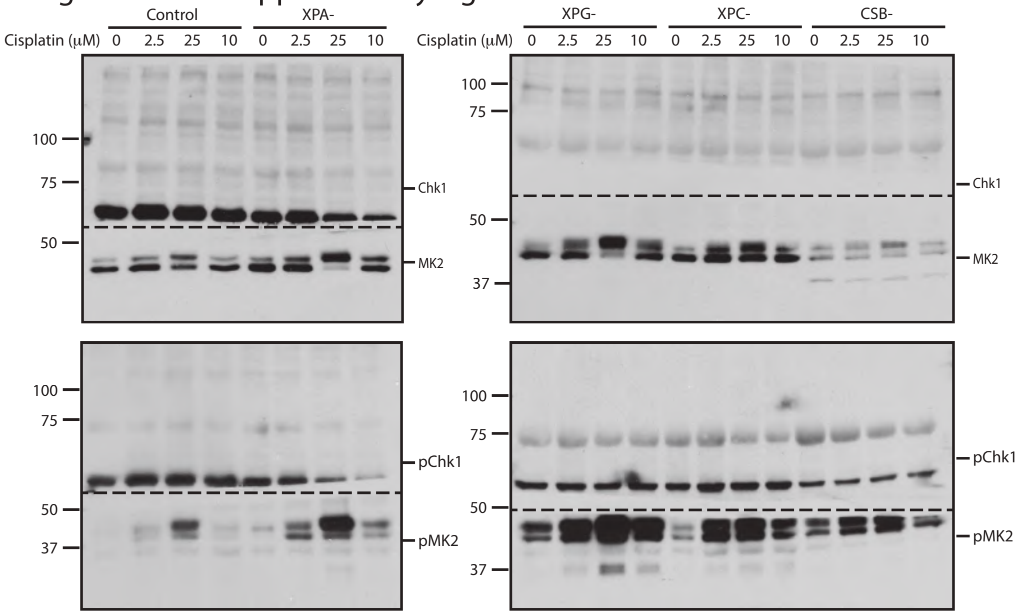

Figure 1C

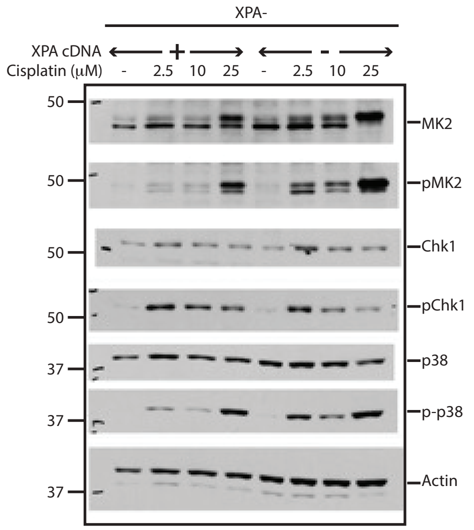

Film overlay onto blots and protein size marked onto film

Figure 1D and Supplementary Figure 1B

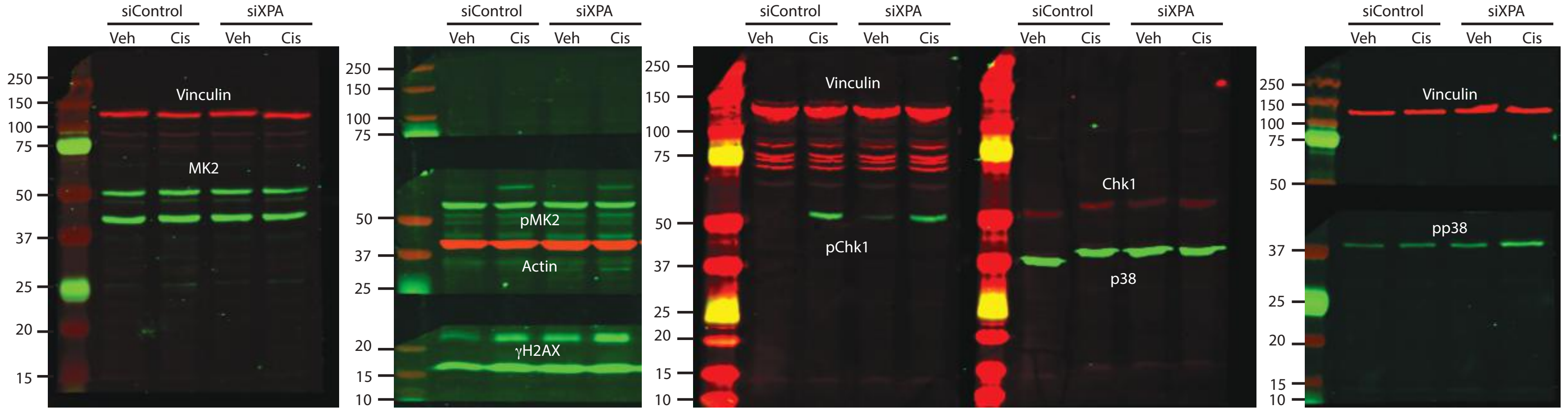

Figure 3C

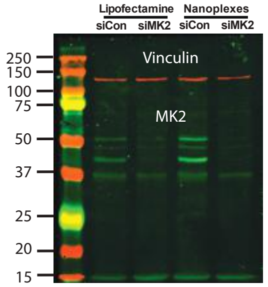

Figure 3G

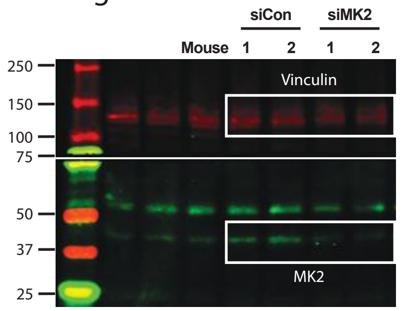

Supplementary Figure 10. Full Western blots

Figure 4B

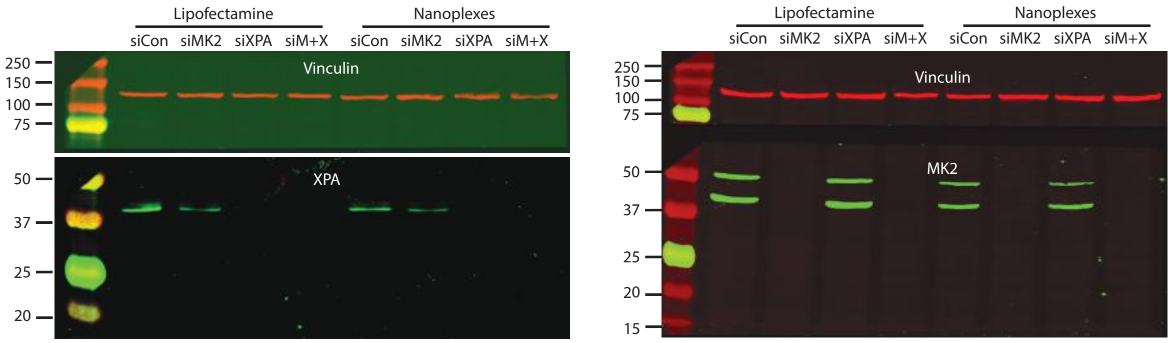

Supplementary Figure 5A

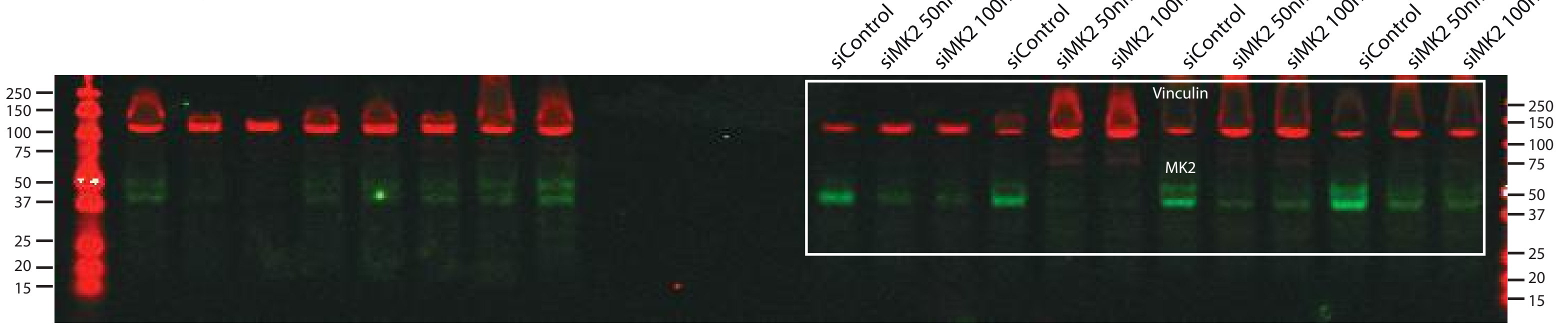

Supplementary Figure 6C

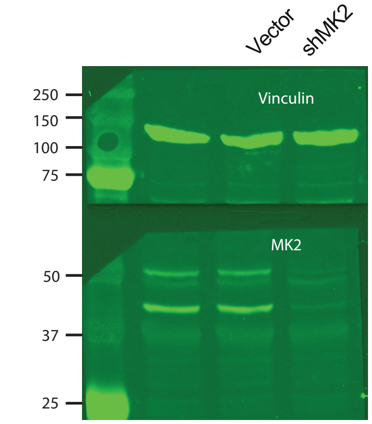

Supplementary Figure 8B

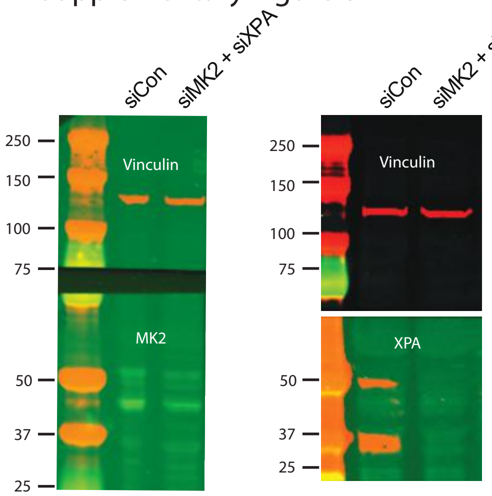

Supplementary Figure 11. Full Western blots
