# Supplementary material for: Enhancing chemotherapy response through augmented synthetic lethality by co-targeting nucleotide excision repair and cell-cycle checkpoints
Source: Nat Commun. 2020 Aug 17;11:4124. doi: 10.1038/s41467-020-17958-z (PMC7431578; doi:10.1038/s41467-020-17958-z)
Supplement: Supplementary file 3 — Reporting Summary [file 41467_2020_17958_MOESM3_ESM.pdf]

nature research

Corresponding author(s): Michael B Yaffe
Paula T Hammond

Last updated by author(s): 1st July 2020

Reporting Summary

Nature Research wishes to improve the reproducibility of the work that we publish. This form provides structure for consistency and transparency in reporting. For further information on Nature Research policies, see our Editorial Policies and the Editorial Policy Checklist.

Statistics

For all statistical analyses, confirm that the following items are present in the figure legend, table legend, main text, or Methods section.

n/a	Confirmed	
☐	☒	The exact sample size (n) for each experimental group/condition, given as a discrete number and unit of measurement
☐	☒	A statement on whether measurements were taken from distinct samples or whether the same sample was measured repeatedly
☐	☒	The statistical test(s) used AND whether they are one- or two-sided<br>*Only common tests should be described solely by name; describe more complex techniques in the Methods section.*
☒	☐	A description of all covariates tested
☒	☐	A description of any assumptions or corrections, such as tests of normality and adjustment for multiple comparisons
☐	☒	A full description of the statistical parameters including central tendency (e.g. means) or other basic estimates (e.g. regression coefficient) AND variation (e.g. standard deviation) or associated estimates of uncertainty (e.g. confidence intervals)
☐	☒	For null hypothesis testing, the test statistic (e.g. F, t, r) with confidence intervals, effect sizes, degrees of freedom and P value noted<br>*Give P values as exact values whenever suitable.*
☒	☐	For Bayesian analysis, information on the choice of priors and Markov chain Monte Carlo settings
☒	☐	For hierarchical and complex designs, identification of the appropriate level for tests and full reporting of outcomes
☒	☐	Estimates of effect sizes (e.g. Cohen's d, Pearson's r), indicating how they were calculated

Our web collection on statistics for biologists contains articles on many of the points above.

Software and code

Policy information about availability of computer code

Data collection	Data was collected from microscopy images using Cell Profiler 3.0.0 and Image J Fiji 1.0.
Data analysis	Microscopy images were analyzed using Cell Profiler 3.0.0 and Image J Fiji 1.0.

For manuscripts utilizing custom algorithms or software that are central to the research but not yet described in published literature, software must be made available to editors and reviewers. We strongly encourage code deposition in a community repository (e.g. GitHub). See the Nature Research guidelines for submitting code & software for further information.

Data

Policy information about availability of data

All manuscripts must include a data availability statement. This statement should provide the following information, where applicable:

- Accession codes, unique identifiers, or web links for publicly available datasets
- A list of figures that have associated raw data
- A description of any restrictions on data availability

The data that support the findings of this study are available from the corresponding authors upon reasonable request. Source data are provided as a Source Data file.

Field-specific reporting

Please select the one below that is the best fit for your research. If you are not sure, read the appropriate sections before making your selection.

☒ Life sciences ☐ Behavioural & social sciences ☐ Ecological, evolutionary & environmental sciences

For a reference copy of the document with all sections, see nature.com/documents/nr-reporting-summary-flat.pdf

Life sciences study design

All studies must disclose on these points even when the disclosure is negative.

Sample size	Power analysis was performed based on preliminary estimates of initial tumor burden and variance, as well as anticipated changes in tumor burden following treatment. The number of animals per group was chosen so as to provide more than 90% statistical power at an alpha level of 0.05 to detect the difference between treatment groups. All tissue culture experiments were performed in triplicates to allow for calculation of standard deviation, standard error of the mean and t-statistics for use in two-tailed Student's t-test.
Data exclusions	No data exclusions except for mice with low tumor burden post inoculation. Exclusion criteria were per-established prior to tumor inocculation.
Replication	All figures presented with error bars were derived from experiments that were successfully repeated in at least 3 replicates.
Randomization	For all mouse experiments, mice were randomized prior to treatment. All other experiments were randomized using randomly assigned plates of cells per condition.
Blinding	For all microscope and histology images, slides were double blinded prior to analysis. Sample were harvested and processed by technicians/scientist without knowledge of hypothesized treatment effects. Results were then properly labeled and assigned once data processing had been completed.

Reporting for specific materials, systems and methods

We require information from authors about some types of materials, experimental systems and methods used in many studies. Here, indicate whether each material, system or method listed is relevant to your study. If you are not sure if a list item applies to your research, read the appropriate section before selecting a response.

Materials & experimental systems

n/a	Involved in the study	
☐	☒	Antibodies
☐	☒	Eukaryotic cell lines
☒	☐	Palaeontology and archaeology
☐	☒	Animals and other organisms
☒	☐	Human research participants
☒	☐	Clinical data
☒	☐	Dual use research of concern

Methods

n/a	Involved in the study	
☒	☐	ChIP-seq
☐	☒	Flow cytometry
☒	☐	MRI-based neuroimaging

Antibodies

Antibodies used	MAPKAPK2, CST, #3042, Lot 3; Phospho-Thr-334 MAPKAPK-2, CST, #3041, Lot 12; Phospho-Ser345 Chk1 (133D3), CST, #2348, Lot 8; Phospho-p38 MAPK (Thr-180/Tyr-182), CST, #9211, Lot 24; Phospho-Histone H2AX (Ser139) (20E3), CST, #9718, Lot 10; Histone H2AX (D17A3), CST, #7631, Lot 1; Chk1, SC, sc-8408, Lot B2307; p38 (A-12), SC, sc-7972; XPA (B-1), SC, sc-28353, Lot F0316;Ki-67, Abcam, ab16667, Lot GR207550-1; anti-cisplatin modified DNA (CP9/19), Abcam, ab103261, Lot GR3192143-1; β-Actin, Sigma Aldrich, A5441, Lot 064M4789V; Gapdh, Sigma Aldrich, G8795, Lot 056M4856V; Vinculin, Sigma, V4505, Lot 128M4874V; CD45-PECy7 (Clone: 30-F11), Thermo (eBioscience), #25-0451-82; CD11b-FITC (Clone: M1/70), Thermo (eBioscience), #11-0112-82; F480-PE (Clone: BM8), Thermo (eBioscience), #12-4801-82; CD3e-APC-Cy7 (Clone: 145-2C11), BioLegend, #100330; IRDye® 800CW Goat anti-Rabbit IgG Secondary Antibody, Li-Cor, 926-32211; IRDye 800CW Goat anti-Mouse IgG (H+L), Li-Cor, 926-32210; IRDye® 680RD Goat anti-Rabbit IgG (H+L), Li-Cor, 926-68071; IRDye® 680RD Goat anti-Mouse IgG (H+L), Li-Cor, 926-68070
Validation	MAPKAPK2, CST, #3042: WB, IF, IP, F, IHC. H, M, R, Mk. 104 citations; CST website antibody validation: 1) Western blot analysis of extracts from untreated or UV treated HeLa and Cos cells show a dual band at expected MW of 47 and 49 kDa. 2) Western blot analysis of shRNA and siRNA knockdown of MAPKAPK2 in the manuscript show expected reduction of both bands.<br><br>Phospho-Thr-334 MAPKAPK-2, CST, #3041: WB, IF. H, M. 49 citations; CST website antibody validation: 1) Western blot analysis of extracts from untreated or UV treated HeLa and Cos cells show the expected increase in p-MK2 in UV treated cells. 2) Immunohistochemical analysis of paraffin-embedded human breast carcinoma show nuclear localization of p-MK2.

Phospho-Ser345 Chk1, CST, #2348: WB, IF. H, M, R, Mk. 355 citations; CST website antibody validation: 1) Western blot analysis of extracts from HeLa, COS, NIH/3T3 and C6 cells, untreated or UV-treated shows single band at expected MW of 56 kDa. 2) Confocal immunofluorescent analysis of C2C12 cells show expected increase p-Chk1 in UV-treated cells. Certificate of Analysis from CST website - https://origin-mediap.cellsignal.com/coa/2348/18/2348-lot-18-coa.pdf

Phospho-p38 MAPK (Thr-180/Tyr-182), CST, #9211: WB, IHC, IP, IF. H, M, R, Dm. 1819 citations; CST website antibody validation: 1) Western blot analysis of extracts from C6 cells, untreated or anisomycin-treated, and NIH/3T3 cells, untreated or UV-treated shows single band at expected MW of 43 kDa. 2) Western blot analysis of extracts from UV-treated NIH/3T3 cells, shows single band at expected MW of 43 kDa. 3) Confocal immunofluorescent analysis of HeLa cells -/+ UV light show absence of staining in untreated cells and nuclear localization of p-p38in treated cells. Certificate of Analysis from CST website - https://origin-mediap.cellsignal.com/coa/9211/25/9211-lot-25-coa.pdf

Phospho-Histone H2AX (Ser139) CST #9718: WB, IF, IHC, ICC-IF. 695 citations, CST website antibody validation: 1) Western blot analysis of extracts from untreated or UV-treated 293 cells shows single band at expected MW of 15 kDa. 2) Immunohisto-chemical analysis of paraffin-embedded HT-29 cells untreated or UV-treated, shows increased nuclear staining in UV treated cells. Certificate of Analysis from CST website - https://media.cellsignal.com/coa/9718/17/9718-lot-17-coa.pdf.

Histone H2AX, CST # 7631: WB, IF, F, IHC, ICC-IF. H, M. 68 citations, CST website antibody validation: 1) Western blot analysis of extracts from various cell lines shows single band at expected MW of 15 kDa. 2) Immunohistochemical analyses of paraffin-embedded human breast, prostate carcinoma shows nuclear staining. Certificate of Analysis from CST website - https://media.cellsignal.com/coa/7631/6/7631-lot-6-coa.pdf.

Chk1, sc-8408: WB, IF, F, PAGE-MAP, ELISA, IP. H, M, C. 573 citations; Chk1 (G-4) is a mouse monoclonal antibody raised against amino acids 1-476 representing full length Chk1 of human origin. CST website antibody validation: 1) Western blot analysis of HeLa cells treated with Chk1 siRNA show Chk1 expression in non-transfected control and reduction in Chk1 siRNA transfected HeLa cells.

p38 (A-12), SC, sc-7972: WB, IP. H, M, R. 287 citations; SC website antibody validation: 1) Western blot analysis of K-562, MDA-MB-231, RAW 264.7, WEHI-231, Jurkat, and NIH/3T3 whole cell lysates show single band at expected Mw of 38kDa. 2) Immunoperoxidase staining of formalin fixed, paraffin-embedded human gall bladder tissue showing cytoplasmic staining of glandular cells. 3) Immunofluorescence staining of methanol-fixed HeLa cells showing cytoplasmic localization.

XPA, SC, sc-28353, Lot F0316: WB, IF, IP. H, Sc. 14 citations; Anti-XPA Antibody (B-1) is a mouse monoclonal IgG1 (kappa light chain) XPA antibody raised against amino acids 1-273 representing full length XPA of human origin and is recommended for detection of XPA of mouse, rat and human origin. SC website antibody validation: 1) Western blot analysis of XPA expression in non-transfected and mouse XPA transfected 293T whole cell lysates show band at expected Mw of 40kDa. 2) Immunoperoxidase staining of formalin fixed, paraffin-embedded human skin tissue showing nuclear staining of fibroblasts, keratinocytes, Langerhans cells and melanocytes.

Ki-67, Abcam ab16667: WB, IF, ICC-IF, ICC, IHC. H, M, Z. 247 citations; Abcam website antibody validation: 1) Ki67 staining in transgenic mouse spinal cord tissue sections (depleted of oligodendrocytes) by Immunohistochemistry (PFA perfusion fixed frozen sections). 2) Fluorescence multiplex immunohistochemical analysis of normal human tonsil tissue (formalin-fixed paraffin-embedded section). 3) Staining Ki67 in wild-type HAP1 cells and Ki67 knockout HAP1 cells show expected loss of staining in knockout cells.

anti-cisplatin modified DNA (CP9/19), Abcam, ab103261, Lot GR3192143-1: F, ICC-IF, ChIP, Dot blot. 24 citations; Rat monoclonal [CP9/19] to Cisplatin modified DNA enables the quantification of cisplatin-induced adducts on DNA. It has also been recently used for isolation of DNA fragments carrying adducts to enhance the sensitivity of subsequent PCR-based analyses and is central to ongoing studies of variation in the nature of cisplatin adducts formed in different cell lines. Abcam website antibody validation: 1) Cisplatin modified DNA in human ovarian cancer cell line A2780 were stained and analyzed by Flow Cytometry. A FITC-conjugated rabbit anti-rat IgG H&L polyclonal (1/200) was used as the secondary antibody. Gating Strategy: FITC positive cells. The sh2 represents the gene knock down cell line which showed positive staining with anti-cisplatin adduct antibody upon cisplatin treatment, whereas the control cells (cis-R, resistant to cisplatin) did not show any staining with anti-cis adduct antibody upon cisplatin treatment.

β-Actin, Millipore Sigma Cat No. A5441: WB, IF, IP, IHC. H, M, R, B. 5604 citations; Monoclonal Anti-β-Actin (mouse IgG1 isotype) is derived from the AC-15 hybridoma produced by the fusion of mouse myeloma cells and splenocytes from an immunized mouse. Immunogen - A slightly modified β-cytoplasmic actin N-terminal peptide, Ac-Asp-Asp-Asp-Ile-Ala-Ala-Leu-Val-Ile-Asp-Asn-Gly-Ser-Gly-Lys, conjugated to KLH. Western blotting of HeLa, JURKAT, COS-7. NIH-3T3, PC-12, RAT2, CHO, MDBK, and MDCK cells shows single band of expected MW of 42 kDa. Certificate of Analysis from Millipore Sigma website - https://www.sigmaaldrich.com/Graphics/COfAInfo/SigmaSAPQM/COFA/A5/A5441/A5441-BULK________079M4799V_.pdf.

Gapdh, Sigma Aldrich G8795: WB, IHC, IF. H, M, R. 731 citations; Western blotting of HeLa, JURKAT, COS-7. NIH-3T3, PC-12, RAT2, CHO, MDBK, and MDCK cells shows single band of expected MW of 37 kDa. Certificate of Analysis from Millipore Sigma website-https://www.sigmaaldrich.com/catalog/CertOfAnalysisPage.do?symbol=G8795&LotNo=056M4856V&brandTest=SIGMA

Vinculin, Sigma V4505, Lot 128M4874V: WB, IF, ICC, IHC. H, M. 247 citations; Monoclonal Anti-Vinculin (mouse IgG1 isotype) is derived from the hybridoma produced by the fusion of mouse myeloma cells and splenocytes from an immunized mouse. Sigma website validation 1) Western blotting of Chicken gizzard cell extract showed band at expected Mw 116kDa. 2) Immunofluorescence staining of mouse adult fibroblast stained at a 1:40 dilution (aqua). Actin (purple) and nuclear DNA (blue) were stained using phalloidin-Alexa Fluor 568 and DAPI, respectively. (Christopher M. Hale, Department of Chemical & Biomolecular Engineering, Johns Hopkins University, Baltimore, MD.) 3) Immunofluorescence-double labeling of an isolated smooth muscle cell (chicken gizzard).

CD45-PECy7 Thermo (eBioscience), #25-0451-82: F, IF, IHC. M. 126 citations; The 30-F11 monoclonal antibody reacts with all isoforms of mouse CD45, also known as Leukocyte Common Antigen (LCA). CD45 is expressed by all hematopoietic cells excluding mature erythrocytes and platelets. The cytoplasmic portion of CD45 has tyrosine phosphatase enzymatic activity and plays an important role in activation of lymphocytes. ThermoFisher website validation. A) Flow-staining of BALB/c splenocytes with 0.06 µg of Rat IgG2b K Isotype Control PE-Cyanine7 or 0.06 µg of Anti-Mouse CD45 PE-Cyanine7. B) Multiple publication using antibody for flow analysis. https://www.thermofisher.com/antibody/product/CD45-Antibody-clone-30-F11-Monoclonal/25-0451-82

CD11b-FITC Thermo (eBioscience), #11-0112-82: F, IHC, IF, WB. M. 248 citations; The M1/70 monoclonal antibody reacts with mouse CD11b, the 165-170 kDa integrin alphaM. CD11b non-covalently associates with CD18 to form alphaMbeta2 integrin (Mac-1) and binds to CD54 (ICAM-1), C3bi, and fibrinogen. Mac-1 is expressed by macrophages, NK cells, granulocytes, activated lymphocytes and mouse B-1 cells in the peritoneal cavity. M1/70 is also cross-reactive to human CD11b, and can be used for the detection of this antigen on human peripheral blood monocytes, granulocytes, and a subset of NK cells. ThermoFisher website validation. A) Flow-staining of mouse bone marrow cells with 0.06 µg of Rat IgG2b K Isotype Control APC or 0.06 µg of Anti-Mouse CD11b APC. Cells in the large scatter population were used for analysis. B) Multiple publication using antibody for flow analysis. https://www.thermofisher.com/antibody/product/CD11b-Antibody-Monoclonal/17-0112-82

F480-PE Thermo (eBioscience), #12-4801-82: F, IF, IHC, ICC. M. 225 citations; The BM8 monoclonal antibody reacts with mouse F4/80 antigen, an approximately 160 kDa surface receptor. It belongs to the EGF-TM7 family of proteins. As such it contains seven EGF-like domains on its extracellular N-terminus, seven transmembrane spanning sequences, and an intracellular C-terminal domain showing homology to other TM7 superfamily members. The F4/80 antigen is expressed by a majority of mature macrophages and is one of the best markers for this population of cells. However, other cell types, such as peritoneal eosinophils, Langerhans cells, and some other dendritic cell subtypes, have been reported to express this antigen as well. Expression of F4/80 commences during early myeloid development in vivo and can be upregulated on BM cells stimulated in vitro with M-CSF. Some populations of macrophages, especially in the lymphoid microenvironment, may be devoid of F4/80. ThermoFisher website validation. A) Flow- staining of mouse resident peritoneal exudate cells with Anti-Mouse CD11b APC and 0.25 µg of Rat IgG2a K Isotype Control FITC or 0.25 µg of Anti-Mouse F4/80 Antigen FIT). Cells in the large scatter population were used for analysis. B) Multiple publication using antibody for flow analysis. https://www.thermofisher.com/antibody/product/F4-80-Antibody-Monoclonal/11-4801-82

CD3e-APC-Cy7(Clone: 145-2C11), BioLegend, #100330; F. M. 56 citations. CD3ε is a 20 kD transmembrane protein, also known as CD3 or T3. It is a member of the Ig superfamily and primarily expressed on T cells, NK-T cells, and at different levels on thymocytes during T cell differentiation. CD3ε forms a TCR complex by associating with the CD3δ, γ and ζ chains, as well as the TCR α/β or γ/δ chains. CD3 plays a critical role in TCR signal transduction, T cell activation, and antigen recognition by binding the peptide/MHC antigen complex. C57BL/6 mouse splenocytes were stained with CD3e (clone 145-2C11) APC/Cyanine7 (filled histogram) or Armenian hamster IgG APC/Cyanine7 isotype control (open histogram).

Eukaryotic cell lines

Policy information about cell lines

Cell line source(s)	NCI-H1299, NCI-H2009, NCI-1563, and HEK 293T were purchased from directly from ATCC. Human fibroblast cell lines GM15876A, GM04312, GM16684, GM00739, and GM16181 were purchased directly from Coriell Institute. KP7B were a gift from Tyler Jacks' laboratory. HCT116 Null cells were a gift from the Vogelstein laboratory.
Authentication	NCI-H1299 and NCI-H2009 were authenticated by STR profiling by Genetica Cell Line Testing, LabCorp. Other cell lines have not been authenticated.
Mycoplasma contamination	All cells were tested negative for mycoplasma using either the universal mycoplasma detection kit from ATCC or by the High Throughput Sciences facilities (Swanson Biotechnology Center, MIT).
Commonly misidentified lines (See ICLAC register)	N/A

Animals and other organisms

Policy information about studies involving animals; ARRIVE guidelines recommended for reporting animal research

Laboratory animals	10-14 week old C57BL6/Jx129-JAE male mice purchased from Jackson Laboratory and housed at 68-72°F; relative humidity at 30-70%; 12/12 dark/light cycle; food (ProLab RMH 3000) and water ad libitum.
Wild animals	Study did not involve wild animals
Field-collected samples	Study did not involve field-collected samples
Ethics oversight	All mouse studies were approved by the MIT Institutional Committee for Animal Care (CAC), and conducted in compliance with the Animal Welfare Act Regulations and other federal statutes relating to animals and experiments involving animals and adheres to the principles set forth in the Guide for the Care and Use of Laboratory Animals, National Research Council, 1996 (Institutional Animal Welfare Assurance #A-3125–01).

Note that full information on the approval of the study protocol must also be provided in the manuscript.

Flow Cytometry

Plots

Confirm that:

☒ The axis labels state the marker and fluorochrome used (e.g. CD4-FITC).

☒ The axis scales are clearly visible. Include numbers along axes only for bottom left plot of group (a 'group' is an analysis of identical markers).

☒ All plots are contour plots with outliers or pseudocolor plots.

☒ A numerical value for number of cells or percentage (with statistics) is provided.

Methodology

Sample preparation	Spleens were isolated from C57BL6/Jx129-JAE mice and mashed through a 40˜m filter. Red blood cells were lysed after incubation with ACK lysis buffer for 5 min and splenocytes were washed with complete growth media (RPMI, 10% FBS, 20mM HEPES, 1mM sodium pyruvate, 0.055mM 2-mercaptoethanol, 2mM L-glutamine, 1x non-essential amino acids and antibiotics). 500,000 splenocytes were co-cultured with 500,000 KP7B tumor cells per replicate per condition and treated for 24h with AF647-siRNA-nanoparticles as indicated. Viable cells were assessed by flow cytometry for uptake of fluorescent siRNA by specific immune populations by co-staining with fluorophore conjugated antibodies for CD45, CD11b, F480 and CD3. CD45+CD11b+AF647+ cells were scored as siRNA+ macrophages, CD45+CD3+AF647+ cells were scored as siRNA+ T-cells and CD45-AF647+ cells were scored as siRNA+ tumor cells.
Instrument	BD LSR Fortessa
Software	Flowjo
Cell population abundance	No sorting was used.
Gating strategy	Please see enclosed Suppl figure for gating strategy.

☒ Tick this box to confirm that a figure exemplifying the gating strategy is provided in the Supplementary Information.
